# Community dialogue meetings among district leaders improved their willingness to receive COVID-19 vaccines in Western Uganda, May 2021

**DOI:** 10.1186/s12889-023-15903-5

**Published:** 2023-05-26

**Authors:** Edirisa Juniour Nsubuga, Arthur G. Fitzmaurice, Allan Komakech, Tom Dias Odoi, Daniel Kadobera, Lilian Bulage, Benon Kwesiga, Peter James Elyanu, Alex Riolexus Ario, Julie R. Harris

**Affiliations:** 1 Uganda Public Health Fellowship Program, Kampala, Uganda; 2 United States Centers for Disease Control and Prevention, Kampala, Uganda; 3 Baylor College of Medicine Children’s Foundation, Kampala, Uganda; 4 Uganda National Institute of Public Health, Kampala, Uganda

**Keywords:** COVID-19 vaccines, COVID-19, District leaders, Pre-post questionnaire, Health behavior, Attitude, Perception, Uganda

## Abstract

**Background:**

Widespread COVID-19 vaccine uptake can facilitate epidemic control. A February 2021 study in Uganda suggested that public vaccine uptake would follow uptake among leaders. In May 2021, Baylor Uganda led community dialogue meetings with district leaders from Western Uganda to promote vaccine uptake. We assessed the effect of these meetings on the leaders’ COVID-19 risk perception, vaccine concerns, perception of vaccine benefits and access, and willingness to receive COVID-19 vaccine.

**Methods:**

All departmental district leaders in the 17 districts in Western Uganda, were invited to the meetings, which lasted approximately four hours. Printed reference materials about COVID-19 and COVID-19 vaccines were provided to attendees at the start of the meetings. The same topics were discussed in all meetings. Before and after the meetings, leaders completed self-administered questionnaires with questions on a five-point Likert Scale about risk perception, vaccine concerns, perceived vaccine benefits, vaccine access, and willingness to receive the vaccine. We analyzed the findings using Wilcoxon’s signed-rank test.

**Results:**

Among 268 attendees, 164 (61%) completed the pre- and post-meeting questionnaires, 56 (21%) declined to complete the questionnaires due to time constraints and 48 (18%) were already vaccinated. Among the 164, the median COVID-19 risk perception scores changed from 3 (neutral) pre-meeting to 5 (strong agreement with being at high risk) post-meeting (*p* < 0.001). Vaccine concern scores reduced, with medians changing from 4 (worried about vaccine side effects) pre-meeting to 2 (not worried) post-meeting (*p* < 0.001). Median scores regarding perceived COVID-19 vaccine benefits changed from 3 (neutral) pre-meeting to 5 (very beneficial) post-meeting (*p* < 0.001). The median scores for perceived vaccine access increased from 3 (neutral) pre-meeting to 5 (very accessible) post-meeting (*p* < 0.001). The median scores for willingness to receive the vaccine changed from 3 (neutral) pre-meeting to 5 (strong willingness) post-meeting (*p* < 0.001).

**Conclusion:**

COVID-19 dialogue meetings led to district leaders’ increased risk perception, reduced concerns, and improvement in perceived vaccine benefits, vaccine access, and willingness to receive the COVID-19 vaccine. These could potentially influence public vaccine uptake if leaders are vaccinated publicly as a result. Broader use of such meetings with leaders could increase vaccine uptake among themselves and the community.

## Background

Attaining high levels of COVID-19 vaccine uptake has been challenging globally [1]. This has been due in part to barriers to vaccine availability and distribution [2, 3], and in part due to vaccine hesitancy [1]. Vaccine hesitancy slows vaccine uptake and may facilitate the emergence of viral variants [4]. In high-income countries, COVID-19 vaccine hesitancy has largely been linked to vaccine safety concerns, political influences, and mistrust and suspicion of vaccine manufacturers [5–7], while in most African countries, hesitancy has been linked to safety and effectiveness concerns [8]. Some vaccine hesitancy has been driven by social media, which has been used to spread misinformation and disinformation about COVID-19 vaccines [9, 10].

One way to improve vaccine uptake is to address misinformation and disinformation through effective risk communication. Risk communication is the real-time exchange of information, advice, and opinions between experts, community leaders, officials, and the people who are at risk and is an integral part of any emergency response [11]. Its major aim is to help the public appreciate the actual risk associated with a disease or event. Risk communication approaches include but are not limited to sensitization over mass media platforms like radios and television stations, utilization of opinion leaders [12], use of peers [12, 13] and dialogues [14, 15]. As a way of addressing misinformation related to COVID-19 and the vaccine and improving uptake, the World Health Organization called upon the global health community in 2020 to implement risk communication interventions about issues related to COVID-19 and the vaccine that engaged with, listened to, informed, and empowered people to make informed decisions to protect themselves and others [10].

The Uganda Ministry of Health began free vaccination against severe acute respiratory syndrome coronavirus 2 (SARS-CoV-2) in March 2021. The Astra-Zeneca vaccine was the only vaccine available in Uganda at the time and required two doses for full vaccination. Vaccination was initially offered to prioritized subpopulations, which included health workers, teachers, adults with comorbidities, and the elderly [16]. However, as of May 1, 2021, only about 355,000 (37%) of 964,000 vaccine doses that were received by the country had been administered, due at least in part to vaccine hesitancy [17, 18].

Identifying locally-appropriate approaches to reduce hesitancy and improve COVID-19 vaccine uptake is critical to epidemic control [19–21]. A February 2021 study in Uganda suggested that public uptake of the COVID-19 vaccine would follow the uptake of vaccination among community leaders [22]. In May 2021, participatory community dialogue meetings about the vaccine were held with district leaders from Western Uganda. The purpose was to promote the uptake of the COVID-19 vaccine among leaders and their communities by improving vaccine knowledge and dispelling myths. We assessed the effect of the meetings on district leaders’ COVID-19 risk perception, COVID-19 vaccine concerns, perceived COVID-19 vaccine benefits, perceived vaccine access, and willingness to receive the COVID-19 vaccine.

## Methods

### Study setting

**Fig. 1:**
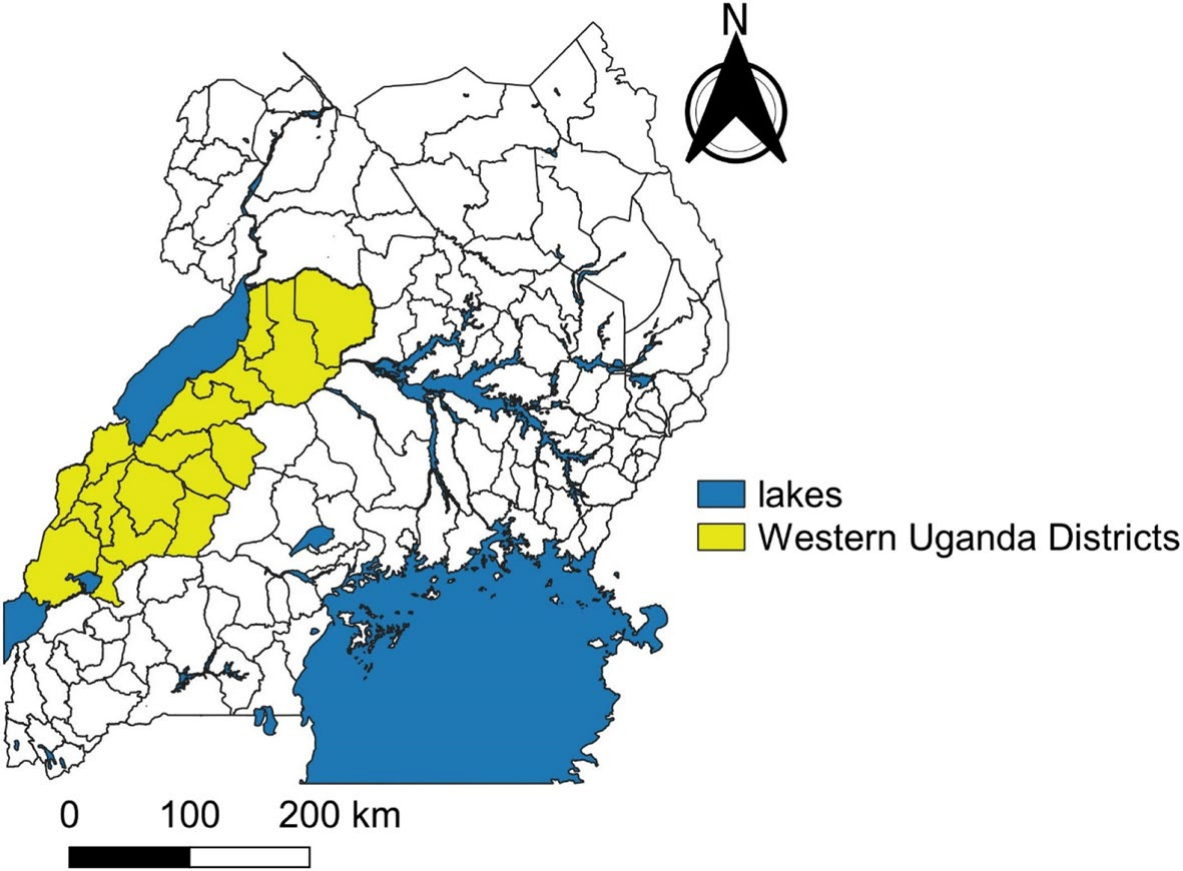
Districts of Western Uganda where community dialogue meetings on district leaders’ willingness to receive the COVID-19 vaccine were conducted, May 2021

We conducted the meetings evaluation in 17 districts of Western Uganda (Fig. 1). As of 2021, an estimated 14% of the Ugandan population resided in the 17 districts [23]. Of the 41,975 COVID-19 cases reported nationally by 1 May 2021, 2,305 (5.5%) were reported in these 17 districts [24]. Despite the availability of free COVID-19 vaccines starting on March 10, 2021, only 20,358 (25%) of the 81,430 doses (including first and second doses) distributed to the 17 districts had been administered by 1 May 2021 [17].

### Study design

We conducted a pre-post evaluation study in which we assessed district leaders’ perceptions of the risk of COVID-19, COVID-19 vaccine concerns, perceived COVID-19 vaccine benefits, perceived vaccine access, and their willingness to receive the COVID-19 vaccine before and after the participatory community dialogue meetings conducted in May 2021. Written reference materials about COVID-19 and COVID-19 vaccines were provided to attendees at the start of the meetings, and the same topics were discussed at all meetings.

#### Community dialogue description

Community dialogue is a forum that brings together people from different sections of society and creates an opportunity to exchange information, perspectives, clarify viewpoints, and develop solutions to issues of interest to society [25–27]. In this study, dialogue participants included political, technical, cultural, and religious leaders.

District leaders from multiple sectors of society were invited to the dialogue meetings in Western Uganda, including political leaders (the district local council five chairpersons, secretaries for health, district councilors, and resident district commissioners), technical leaders (chief administrative officers and district heads of departments such as health, education, planning, production, works, administration, human resources, and finance), district religious leaders from all prevalent faiths in Uganda, and cultural leaders. One community dialogue meeting was held in each of the 17 districts, and 14–21 district leaders from each district participated in the meetings. On average, each meeting lasted approximately four hours. A total of 268 district leaders from 17 districts participated in the meetings and were invited to participate in the evaluation assessment (Fig. 1).

After arrival and registration, participants completed a pre-meeting assessment questionnaire, followed by opening prayers, self-introductions, brief remarks by Baylor Uganda staff, and opening remarks by district health officers. Meetings were chaired and facilitated by district health educators (DHEs), who presented general information about COVID-19 (what it is, signs and symptoms, who is at risk of getting COVID-19, how one can be protected from contracting it), the types and availability of different COVID-19 vaccines in circulation, how the vaccines work, how they were developed, and why they were developed in a short time. The presentations were based on recent trainings for the DHEs given by Baylor Uganda on these topics.

The DHEs also talked about which type of COVID-19 vaccines are given to Ugandans, why those were the available vaccines, who were eligible to receive those vaccines (essential workers including the district leaders), and why they were eligible. Other topics included vaccine availability, cost (emphasizing that vaccines were free of charge), physical access to vaccines, administration, safety, effectiveness against COVID-19 infection and severe forms of COVID-19, common vaccine side effects, risks of serious reactions, and how to deal with them. DHEs also discussed personal health risks from COVID-19 and the consequences of not vaccinating.

After their presentations, DHEs encouraged participants to ask questions, raise points of concern, and discuss the answers amongst themselves with the guidance of DHEs and other technical professionals. After issues and concerns about the vaccines were discussed to participant’s satisfaction, DHEs summarized key messages and closed the meetings. Closing activities included the development of an action plan on how each participant would disseminate the information they received from the meeting to the community to promote vaccine uptake. Two trained staff members of Baylor Uganda served as rapporteurs during each meeting. Each leader was invited to complete the same evaluation questionnaire that was administered at the beginning of the meeting.

#### Questionnaire

We performed the assessment based on three of the most prominent health behavior theory constructs: the health belief model [28], the theory of planned behavior [29], and the extended parallel process model [30]. We used these theories to develop self-administered questionnaires to assess COVID-19 risk perception, vaccine concerns, perceived vaccine benefits, perceived vaccine access, and willingness to receive a COVID-19 vaccine. For the five-point Likert scale questions, participants indicated 1 (strongly disagree), 2 (disagree), 3 (neither disagree nor agree), 4 (agree), or 5 (strongly agree).

### Study variables

In the questionnaire, we also asked about sociodemographic factors, including the highest education level attained, presence of children aged < 5 years or persons ≥ 60 years at home, and district of work. We organized the questions into five categories: COVID-19 risk perception (subcategories: perceived susceptibility, perceived severity), COVID-19 vaccine concerns, perceived COVID-19 vaccine benefits (subcategories: perceived individual benefits, perceived community benefits/altruism), perceived COVID-19 vaccine access, and willingness to receive a COVID-19 vaccine. We constructed composite scores by summing scores from multiple questions within a category or subcategory.

### Data analysis

We analyzed the data using STATA Version 14.0. We described sociodemographic factors using frequencies and percentages. Likert scale data were ordinal and not normally distributed when tested for normality using the Shapiro–Wilk tests, so we used the nonparametric Wilcoxon signed-rank test to assess differences between pre- and post-dialogue scores within each category and subcategory [31, 32]. We used Wilcoxon’s signed-rank test instead of the sign test because it has more statistical power [33]. Wilcoxon’s signed-rank test ranks the degree of change between the paired scores in addition to considering the degree of change measured by the sign test, providing more information for analysis [33].

To calculate the magnitude of the effect of the community dialogue meetings on each category and subcategory, we used Cliff’s delta measure (Cliff’s dominance measure), which is the accepted measure of effect size for the Wilcoxon signed-rank test [34, 35], to calculate the effect sizes (*r*) of the changes [36]. It is obtained by subtracting the ratio of the negative rank-sum to the total rank-sum from that of the positive rank-sum to the total rank-sum [37, 38]. The effect size ranges from 0 to 1, with 0 indicating that the groups are statistically equal and 1 implying that one group significantly dominates [37, 38]. We graded the effect size as small (*r* = 0.1–0.3), medium (*r* = 0.4–0.5), and large (*r* = 0.6–1.0) for both positive and negative changes [36]. We also reported median frequencies, percentages, medians, and first and third quartiles, which we used to calculate interquartile ranges (IQRs) for both pre- and post-meeting assessments.

We also conducted Spearman’s correlation between willingness to receive the COVID-19 vaccine and each category and subcategory to assess factors associated with willingness to receive COVID-19 vaccine. We only tested for correlations pre-meeting when willingness was normally distributed and before social desirability bias was introduced by the meeting. The Spearman correlation evaluates the monotonic relationship between two ordinal variables [39]. We also performed logistic regression to assess whether COVID-19 vaccine willingness was associated with the presence of children aged < 5 years or ≥ 60 years at home.

## Results

### Final evaluation sample size

Among the 268 community dialogue meeting attendees, 164 (61%) filled out both pre- and post-meeting assessments. Forty-eight (18%) who had already been vaccinated and 56 (21%) who completed only the pre-meeting assessment due to time constraints were excluded from the analysis.

### Sociodemographic characteristics of community dialogue participants

In total, 150 (92%) of the 164 district leaders who participated in the study had attained either a tertiary or university education; the rest had attained secondary or primary education. Most (118, 72%) were men (Table 1).

**Table 1:** Sociodemographic characteristics of community dialogue participants, Western Uganda, May 2021 (*N* = 164)

Variable	Frequency (n)	Percent
**Education**		
Primary	2	1
Secondary	12	7
Tertiary/University	150	92
**Having children aged < 5 years in the household**		
No	56	34
Yes	108	66
**Having persons aged ≥ 60 years in the household**		
No	114	70
Yes	50	30
**Sex**		
Female	46	28
Male	118	72
**Was a frontline worker during COVID-19 response**^a^		
No	48	29
Yes	116	71

^a^A frontline worker who worked during the COVID-19 response, e.g., a health worker and a COVID-19 district task force member

### COVID-19 risk perception, vaccine concerns, perceived vaccine benefits, perceived vaccine access, and willingness to receive the COVID-19 vaccine

**Fig. 2:**
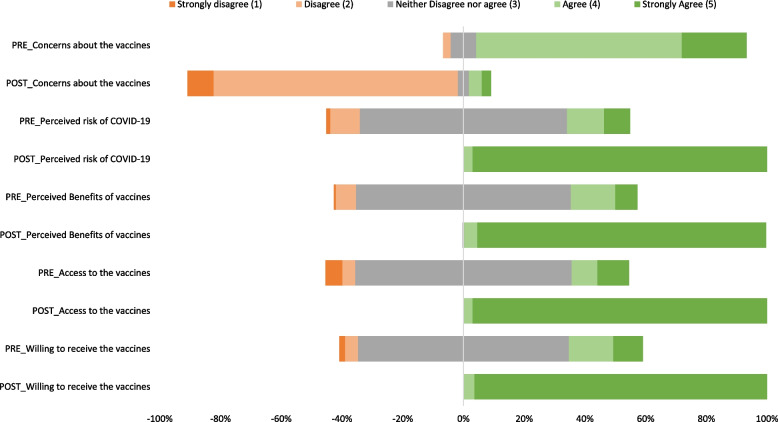
Summary of the effect of community dialogue meetings on district leaders’ COVID-19 risk perception, COVID-19 vaccine concerns, perceived COVID-19 vaccine benefits, perceived COVID-19 vaccine access, and willingness to receive the COVID-19 vaccine, Western Uganda, May 2021 (*N* = 164)

The meetings were associated with positive changes in leaders’ perception of the risk of COVID-19 (Wilcoxon’s signed-rank test *p* < 0.001, Cliff’s delta measure *r* = 0.995), perceived COVID-19 vaccine benefits (*p* < 0.001, *r* = 0.995), perceived COVID-19 vaccine access (*p* < 0.001, *r* = 0.996), and willingness to receive the COVID-19 vaccine (*p* < 0.001, *r* = 0.995). The meetings were also associated with a reduction in leaders’ concerns about COVID-19 vaccine safety and side effects (*p* < 0.001; *r* = -0.960) (Table 2, Fig. 2).

**Table 2:** Effect of community dialogue meetings on district leaders’ COVID-19 risk perception, COVID-19 vaccine concerns, perceived COVID-19 vaccine benefits, perceived COVID-19 vaccine access, and willingness to receive the COVID-19 vaccine, Western Uganda, May 2021 (*N* = 164)

**Variable**	**Median (IQR)**		***p*-value**^a^	**Effect Size (*r*)**^b^
	**Pre-dialogue**	**Post-dialogue**		
**COVID-19 risk perception**	**3 (3,3)**	**5 (5,5)**	**< 0.001**	**0.995**
**Perceived susceptibility to COVID-19**	**3 (3,3)**	**5 (5,5)**	**< 0.001**	**0.993**
I am at risk of getting COVID-19	3 (3,3)	5 (5,5)	< 0.001	0.987
I will likely get COVID-19	3 (3,3)	5 (5,5)	< 0.001	0.996
I may get COVID-19	3 (3,3)	5 (5,5)	< 0.001	0.985
COVID-19 is real	3 (3,3)	5 (5,5)	< 0.001	1.000
**Perceived severity of COVID-19**	**3 (3,4)**	**5 (5,5)**	**< 0.001**	**1.000**
I believe that COVID-19 has serious negative consequences	3 (3,4)	5 (5,5)	< 0.001	0.984
I believe that COVID-19 is a severe health problem	3 (3,4)	5 (5,5)	< 0.001	0.985
I believe that COVID-19 can be very harmful to persons infected with it	3 (3,3)	5 (5,5)	< 0.001	0.996
**COVID-19 vaccine concerns**	**4 (4,4)**	**2 (2,2)**	**< 0.001**	**-0.960**
I have concerns about the possible side effects of COVID-19 vaccines	4 (4,5)	2 (2,2)	< 0.001	-0.959
There has been limited research done on the COVID-19 vaccines	4 (4,5)	2 (2,2)	< 0.001	-0.941
I have concerns about the safety of the COVID-19 vaccines	4 (4,4)	2 (2,2)	< 0.001	-0.955
**COVID-19 vaccines benefits**	**3 (3,3)**	**5 (5,5)**	**< 0.001**	**0.995**
**Perceived individual benefits of vaccines**	**3 (3,3)**	**5 (5,5)**	**< 0.001**	**0.997**
Getting vaccinated is for my benefit	3 (3,3)	5 (5,5)	< 0.001	0.987
If I get the vaccine, I will be less likely to get COVID-19	3 (3,3)	5 (5,5)	< 0.001	0.993
COVID-19 vaccines can prevent COVID-19	3 (3,3)	5 (5,5)	< 0.001	1.000
**Perceived community benefits of COVID-19 vaccines**	**3 (3,4)**	**5 (5,5)**	**< 0.001**	**0.995**
Having myself vaccinated against COID-19 is beneficial for the health of others in my community	3 (3,4)	5 (5,5)	< 0.001	0.995
COVID-19 vaccines protect the health of my community	3 (3,4)	5 (5,5)	< 0.001	0.995
**COVID-19 vaccine access**	**3 (3,3)**	**5 (5,5)**	**< 0.001**	**0.996**
As soon as I am eligible to receive the vaccine, I will be able to get vaccinated to prevent contracting the disease	3 (3,3)	5 (5,5)	< 0.001	0.996
When I am eligible to receive the vaccine, it will be easy for me to get it to protect myself from the disease	3 (3,3)	5 (5,5)	< 0.001	0.996
**Willingness to receive COVID-19 vaccines**	**3 (3,3)**	**5 (5,5)**	**< 0.001**	**0.995**
I am thinking of getting the vaccine	3 (3,4)	5 (5,5)	< 0.001	1.000
I am prepared to receive the vaccine	3 (3,3)	5 (5,5)	< 0.001	1.000
I will get vaccinated if a health worker offers me the vaccine	3 (3,3)	5 (5,5)	< 0.001	0.993
I will get vaccinated for COVID-19	3 (3,3)	5 (5,5)	< 0.001	0.996

Median IQR of 1 = strongly disagree, 2 = disagree, 3 = neither disagree nor agree, 4 = agree, or 5 = strongly agree

^a^*p*-values as per Wilcoxon’s signed-rank test

^b^Effect size (r) as per Cliff’s delta measure

### Correlations between willingness to receive the COVID-19 vaccine and the other categories and subcategories before the meetings

Pre-assessment, willingness to receive the COVID-19 vaccine was negatively correlated with COVID-19 vaccine concerns (Spearman’s rho (*ρ*) = -0.31, *p* = 0.001) and positively correlated with COVID-19 risk perception (*ρ* = 0.66, *p* < 0.001), perceived susceptibility (*ρ* = 0.70, *p* < 0.001), and perceived COVID-19 severity (*ρ* = 0.69, *p* < 0.001). It was also positively correlated with perceived vaccine benefits (*ρ* = 0.72, *p* < 0.001), individual benefits (*ρ* = 0.75, *p* < 0.001), and community benefits (*ρ* = 0.68, *p* < 0.001), as well as perceived vaccine access (*ρ* = 0.81, *p* < 0.001) (Table 3).

**Table 3:** Spearman’s correlation coefficients (*ρ*) between willingness to receive the COVID-19 vaccine before the meetings and COVID-19 risk perception, vaccine concerns, vaccine benefits, and access before the meetings, Western Uganda, May 2021

**Variable**	**Spearman’s rho (ρ)**	***p*-value**^a^
**COVID-19 risk perception**	**0.66**	**< 0.001**
Perceived susceptibility to COVID-19	0.70	< 0.001
Perceived severity of COVID-19	0.69	< 0.001
**COVID-19 vaccine concerns**	**-0.31**	**< 0.001**
**COVID-19 vaccine benefits**	**0.72**	**< 0.001**
Perceived individual benefits	0.75	< 0.001
Perceived community benefits	0.68	< 0.001
**COVID-19 vaccine access**	**0.81**	**< 0.001**

^a^*p*-value as per Spearman’s correlation

## Discussion

Following a study in Uganda in early 2021 that suggested that community vaccine uptake would follow community leader vaccine uptake [22], we evaluated the use of dialogue meetings with district leaders to improve their willingness to receive COVID-19 vaccine. Community dialogue meetings with local leaders about the COVID-19 vaccine and the risk of COVID-19 disease in Western Uganda led to reduced concerns about COVID-19 vaccine safety and side effects, improvement in perceived vaccine benefits and access, increased COVID-19 risk perception, and increases in leaders’ willingness to receive COVID-19 vaccines. Willingness to receive COVID-19 vaccines was negatively correlated with COVID-19 vaccine concerns and positively correlated with COVID-19 risk perception, perceived benefits, and perceived vaccine access.

During the dialogue meetings, personal health risks from COVID-19, vaccine effectiveness against COVID-19 infection and severe forms of COVID-19, and the consequences of not being vaccinated were discussed in depth. Additionally, access to vaccines, including knowing where to obtain vaccines and having economic and physical access to the vaccine, were also discussed. Designated vaccination areas were mentioned, and it was emphasized that the COVID-19 vaccines were free of charge and were to be given to prioritized subpopulations, such as the district leaders, first. We found that district leaders’ COVID-19 risk perception increased after the dialogue meetings, as did their perceptions about access to and the benefits of the COVID-19 vaccine. Having a perception of risk associated with contracting COVID-19 is necessary for interest in taking the vaccine; people are unlikely to want to be vaccinated against something for which they feel they have little risk. Indeed, in our study, we found a positive association between COVID-19 risk perception and willingness to receive the vaccine, as well as between perceived vaccine access and benefits and willingness to receive the vaccine. Similarly, a study done in the US showed that messages emphasizing the personal health risks of COVID-19 and collective health consequences of not vaccinating significantly increased Americans’ perceived benefits and hence intentions to vaccinate [40]. In another study among mainland Chinese university students, students’ knowledge of the COVID-19 vaccine and risk perception of COVID-19 positively influenced their attitude toward the uptake of a COVID-19 vaccine [41]. Our findings are also consistent with those from a study carried out among Chinese factory workers which showed that perception of easy access to COVID-19 vaccines increased with a decrease in the cost of the vaccines [20].

However, the true level of risk associated with the vaccine itself also needed to be addressed. In Uganda, as in most African countries, the main concerns about COVID-19 vaccines were related to both vaccine effectiveness and vaccine safety: primarily the short time in which COVID-19 vaccines were developed, concerns about insufficient testing of vaccines, and potential side effects [8]. Our discussions with community leaders included a major component about vaccine safety and data supporting vaccine effectiveness and resulted in a decrease in district leaders’ concerns about the safety and side effects of COVID-19 vaccines after the meetings. A study carried out in the United States by Chu et al*.* showed that confidence in vaccine safety, increased disease risk perception, and increased perceived vaccine benefits were all major factors associated with increased willingness to receive the COVID-19 vaccines [21].

Previous studies in Malawi, Tanzania, Ethiopia, South Africa, Uganda, and the United States have shown that community dialogue meetings can play vital roles in supporting or opposing health service utilization through the mobilization of community members [42–44]. For COVID-19 vaccines specifically, open and transparent dialogue and communication about uncertainty and risks, including around the safety and benefits of COVID-19 vaccination provided during community dialogue meetings, can ensure a shared understanding of the benefits of COVID-19 vaccination to each individual and the community as a whole [45, 46]. Our findings are consistent with multiple other studies that have shown that the use of dialogues between health providers and communities and listening to and addressing people’s concerns about vaccine safety can be used to reduce concerns and distrust and improve interest in vaccination [47–49]. Our findings reiterate the need for actively promoting the effectiveness and importance of vaccination, while addressing concerns about vaccine safety in the public [50] when handling future outbreaks and emergencies.

We found community dialogue meetings to effectively reduce concerns about COVID-19 vaccine safety and side effects, improve perceptions about vaccine benefits and access, increase COVID-19 risk perception, and improve willingness to receive COVID-19 vaccines. Other forms of dialogues that can be used could include social media, such as Twitter or Facebook dialogues, and one-on-one dialogue meetings [9, 51, 52]. While most approaches to risk communication during emergencies – such as radio and television talk shows—often use a one-directional approach [52, 53], these methods don’t allow for the public to share their concerns, and for these concerns to be addressed. Community dialogue meetings are two-way processes that involve clear message delivery by health experts to the community [53]. The two-way communication allows the public to share their concerns, and for these concerns to be addressed [52]. If done well, community dialogue meetings can facilitate public trust, confidence, and, importantly, compliance with the recommended behaviors [52, 54–57]. Accordingly, when handling pandemics and emergencies in future, community dialogue meetings can be prioritized as vital risk communication methods.

### Study limitations

Although we report changes in community leaders’ perceived risk, benefits, access, safety concerns and willingness to receive COVID-19 vaccines after the meetings, we do not know if the theoretical changes eventually led to vaccine uptake among leaders or among the general public.

## Conclusions

The community leader dialogue meetings reported here led to district leaders’ increased COVID-19 risk perception, reduced concerns of COVID-19 vaccine safety and side effects, and improvement in perceived COVID-19 vaccine benefits, perceived vaccine access, and willingness to receive the COVID-19 vaccine. These improvements might influence public uptake of the COVID-19 vaccines if leaders get vaccinated publicly and share their vaccination status and what they learned during dialogue meetings. Broader use of community leader dialogue meetings as a way of reducing COVID-19 vaccine hesitancy and increasing uptake, in addition to other methods like the use of opinion leaders to encourage vaccine acceptability and mass media platforms such as radio and television talk shows, may be considered. Scaling up community dialogue meetings to involve community members might also increase vaccine uptake.

## Data Availability

The datasets upon which our findings are based belong to the Uganda Public Health Fellowship Program. For confidentiality reasons, the datasets are not publicly available. However, the datasets can be made available upon reasonable request from the corresponding author and with permission from the Uganda Public Health Fellowship Program.

## Abbreviations

COVID-19: Coronavirus Disease 2019
DHE: District Health Educator
IQR: Interquartile Range
